# MSCs overexpressing GDNF restores brain structure and neurological function in rats with intracerebral hemorrhage

**DOI:** 10.1186/s43556-023-00159-7

**Published:** 2023-11-27

**Authors:** Xiaoqian Jiang, Ling Zhou, Zihuan Sun, Bingqing Xie, Heng Lin, Xiaoqing Gao, Li Deng, Chaoxian Yang

**Affiliations:** 1 Department of Anatomy, College of Basic Medicine, Southwest Medical University, Luzhou, 646000 Sichuan China; 2 Clinical Skills Center, the Affiliated Hospital of Southwest Medical University, Luzhou, 646000 Sichuan China; 3 Laboratory of Neurological Diseases and Brain Function, the Affiliated Hospital of Southwest Medical University, Luzhou, 646000 Sichuan China; 4 Institute of Epigenetics and Brain Science, Southwest Medical University, Luzhou, 646000 Sichuan China; 5 Department of Neurobiology, Preclinical Medicine Research Center, Southwest Medical University, Luzhou, 646000 Sichuan China

**Keywords:** Intracerebral hemorrhage, Mesenchymal stem cells, Glial cell-derived neurotrophic factor, Synaptic plasticity, Neurological recovery

## Abstract

Mesenchymal stem cells (MSCs) have been applied in transplantation to treat intracerebral hemorrhage (ICH) but with limited efficacy. Accumulated evidence has shown that glial cell-derived neurotrophic factor (GDNF) plays a crucial part in neuronal protection and functional recovery of the brain after ICH; however, GDNF has difficulty crossing the blood–brain barrier, which limits its application. In this study, we investigated the influences of MSCs overexpressing GDNF (MSCs/GDNF) on the brain structure as well as gait of rats after ICH and explored the possible mechanisms. We found that cell transplantation could reverse the neurological dysfunction and brain damage caused by ICH to a certain extent, and MSCs/GDNF transplantation was superior to MSCs transplantation. Moreover, Transplantation of MSCs overexpressing GDNF effectively reduced the volume of bleeding foci and increased the level of glucose uptake in rats with ICH, which could be related to improving mitochondrial quality. Furthermore, GDNF produced by transplanted MSCs/GDNF further inhibited neuroinflammation, improved mitochondrial quality and function, promoted angiogenesis and the survival of neurons and oligodendrocytes, and enhanced synaptic plasticity in ICH rats when compared with simple MSC transplantation. Overall, our data indicate that GDNF overexpression heightens the curative effect of MSC implantation in treating rats following ICH.

## Introduction

Intracerebral hemorrhage (ICH), the second most common subtype of stroke, is a critical cerebrovascular disease that usually causes catastrophic brain damage and leads to severe disability or death. The treatment strategy for ICH may include medical and surgical therapies to prevent the bleeding, clear the clot, and alleviate the pressure on the brain, but the prognosis is poor [1–3]. In recent years, the mortality and disability rates of ICH have remained high, and there is still a lack of effective treatments [4]. After ICH, the local microenvironment deteriorates, leading to massive death of neurons and oligodendrocytes. These changes are a vital factor of the disability and death in patients [5, 6]. Therefore, improving the microenvironment, reducing cell death, and enhancing synaptic plasticity are the basis for the neurological recovery of patients.

Many studies suggest that stem cell therapy is an up-and-coming treatment strategy that can improve the prognosis of ICH [7, 8]. Among the various types of stem cells that may be useful for the treatment of ICH, mesenchymal stem cells (MSCs) possess great medical value owing to their self-renewal, multi-lineage differentiation properties, low immune responsiveness, easy to obtain, and lack of significant ethical concerns [9–11]. Previous studies indicated that MSC transplantation therapy improved neurological functions, reduced lesion volume, and enhanced neural network reconstruction via various mechanisms, including promoting neurogenesis and angiogenesis, and inhibiting apoptosis and inflammation in experimental ICH models [6, 7]. Clinical reports demonstrated that transplantation of MSCs improved neurological outcomes in post-hemorrhagic stroke patients [12, 13]. Nevertheless, it remains difficult for a simple MSC transplantation to achieve the desired effect [14].

It is widely known that glial cell-derived neurotrophic factor (GDNF) exerts a robust neuroprotective effect in various neurological diseases. The administration of lentiviral GDNF could reduce post-ischemic neurological deficits and neuronal injury, and promote brain remodeling and neuroplasticity via regulating guidance molecules and axonal growth inhibitors [15]. GDNF could also protect the hippocampus against excitotoxic damage in rats after stroke [16]. Pretreatment with GDNF significantly prevented NO release and reduced cortical infarction during ischemia–reperfusion injury in rats [17]. Studies have shown that GDNF exerts neuroprotective effects by acting on multiple physiological links. However, the local application of GDNF to reduce the infarct area in ischemic stroke rats was time-dependent and the therapeutic time window was short [18]. Therefore, gene therapy of GDNF is a better way for its clinical application.

Multiple effects may occur when the GDNF gene is transfected into MSCs by gene recombination technology, and the genetically modified MSCs (MSCs/GDNF) are transplanted into the lesion or injury site of the nervous system. Our previous experiments showed that MSCs overexpressing the GDNF gene ameliorated functional deficiency, downscaled lesion volumes, and increased the expression of microtubule associated protein 2 (MAP2) and neuron-specific enolase (NSE) proteins in ICH rats [19, 20]. However, many aspects of the effect and mechanism of MSCs/GDNF on ICH are still obscure. In this study, we investigated whether implantation of MSCs overexpressing GDNF could improve gait and brain structure in hemorrhagic apoplexy, and inquired into the possible mechanisms involved. Our data show that compared with MSCs transplantation, MSCs/GDNF transplantation can improve brain structure and key gait parameters reflecting the overall motor ability of animals whilst reducing neuroinflammation.

## Results

### MSCs/GDNF transplantation improves neurological function

**Fig. 1:**
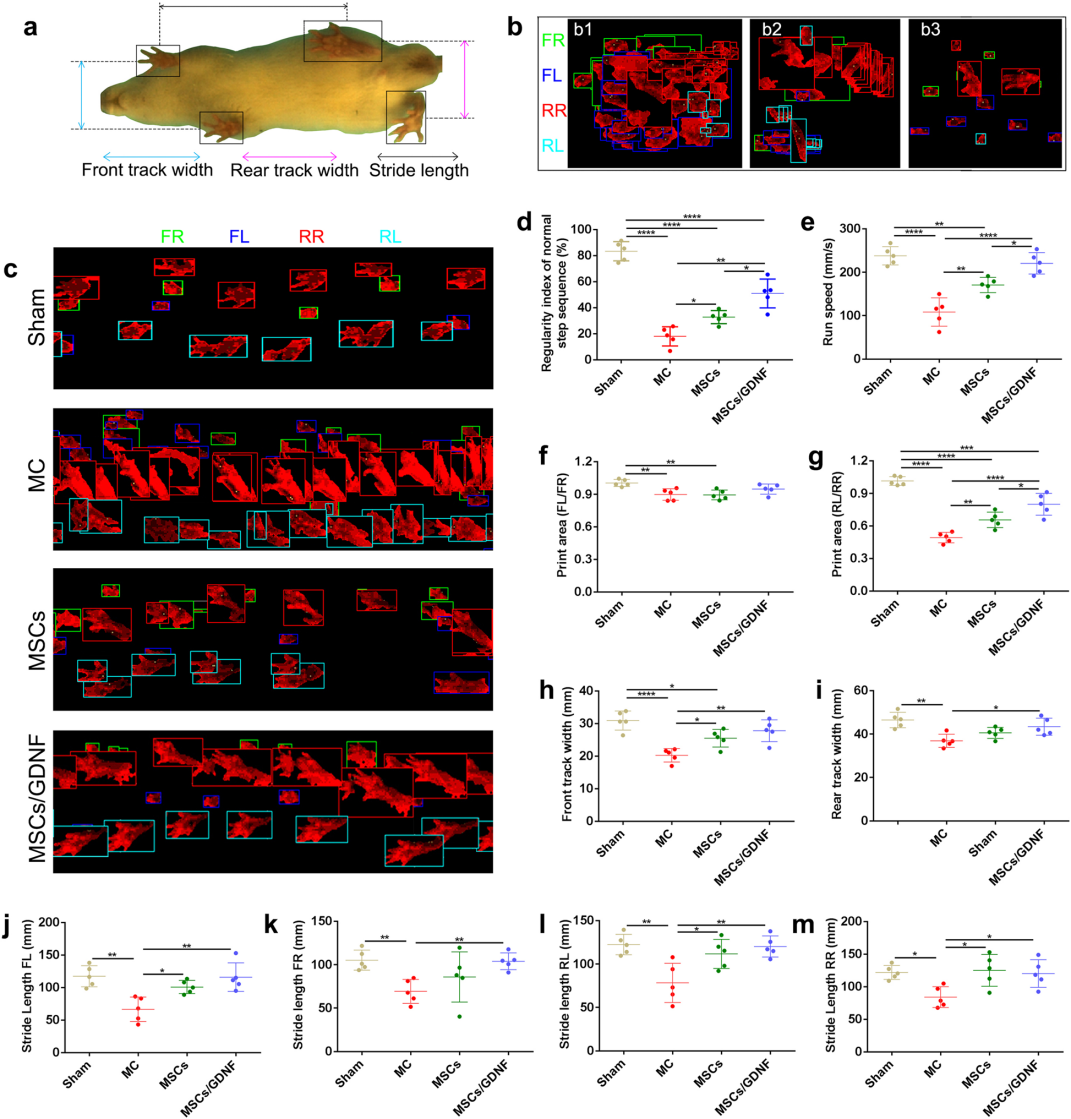
Gait analysis using TreadScan to evaluate motor ability of rats. **a** Representative rat moving image. **b** Representative paw prints of motor dysfunction symptoms in rats after ICH (b1: rotation; b2: slow movements; b3: smaller footprints). **c** Representative paw prints of rats in different groups. **d-m** Gait parameters of the rats in different groups were dealt with GAIT SCAN analysis software (*n* = 5 per group). The parameters include regularity index of normal step sequence, run speed, print area, track width, and stride length. FL, FR, RL and RR represent front left, front right, rear left and rear right paws, respectively. *P < 0.05, **P < 0.01, ***P < 0.001, and ****P < 0.0001

**Fig. 2:**
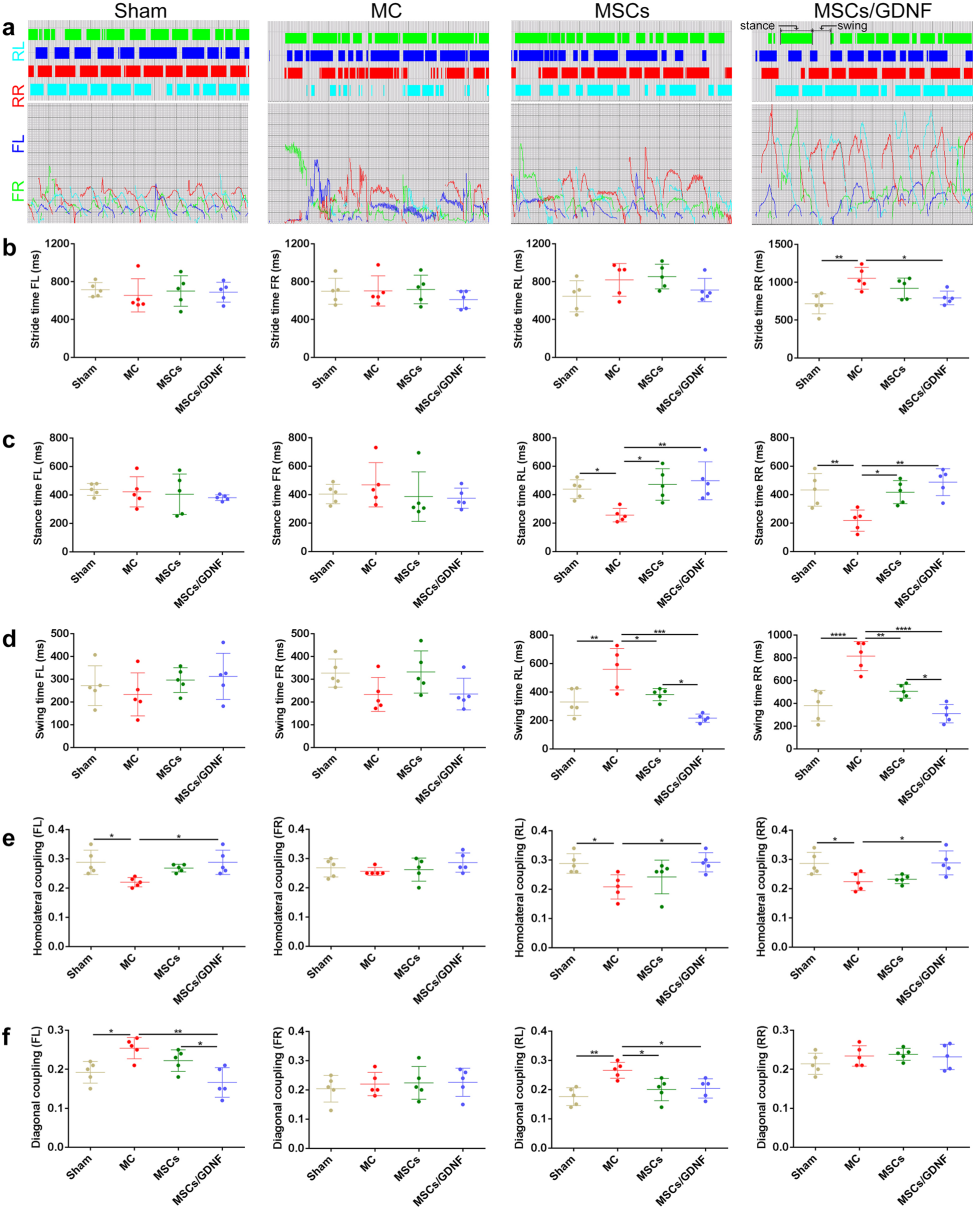
Motor function measured by TreadScan. **a** Representative strides (including stance and swing) of the rats in different groups. **b**-**f** Gait parameters of the rats in different groups were dealt with GAIT SCAN analysis software (*n* = 5 per group). The parameters include stride time, stance time, swing time, homolateral coupling, and diagonal coupling. FL, FR, RL and RR represent front left, front right, rear left and rear right paws, respectively. *P < 0.05, **P < 0.01, and ****P < 0.0001

ICH causes neurological dysfunction. To comprehensively and objectively evaluate the effect of MSCs overexpressing GDNF on the neurological function in rats with ICH, we adopted the TreadScan gait analysis system. Figure 1a shows the rat moving image. After ICH, the rats presented with neurological symptoms such as rotation, slow movements (overlapping footprints), and smaller footprints (Fig. 1b). Gait analysis displayed apparent alteration in some parameters in ICH rats at day 7 after cell transplantation (Figs. 1 and 2). The cell transplantation treatment improved the rate of normal step sequence, run speed, print area (rear left paw (RL)/rear right paw (RR)), front track width, stride length (except for front right paw (FR)), stance time (RR and RL), but reduced swing time (RR and RL) and diagonal coupling (RL) (*p* < 0.05). Furthermore, the MSCs/GDNF group showed a better therapeutic effect than the MSCs group on the rate of normal step sequence, run speed, print area (RL/RR), swing time (RR and RL), and diagonal coupling (FL) (*p* < 0.05). For some parameters, although there were no significant differences between the MSCs/GDNF group and the MSCs group, and the MSCs group and the MC group, the MSCs/GDNF group increased the rear track width and stride length (FR), and decreased the stride time (RR) and homolateral coupling (FL, RR and RL) when compared with the MC group (*p* < 0.05). It is worth noting that the rate of normal step sequence, run speed, and limb coordination (including homolateral coupling and diagonal coupling) are vital indicators that reflect the overall motor ability of animals. Therefore, the above results suggest that MSCs/GDNF transplantation can better improve the neurobehavioral function of rats with ICH than MSCs transplantation.

### MSCs/GDNF transplantation improves brain tissue structure and glycometabolism

**Fig. 3:**
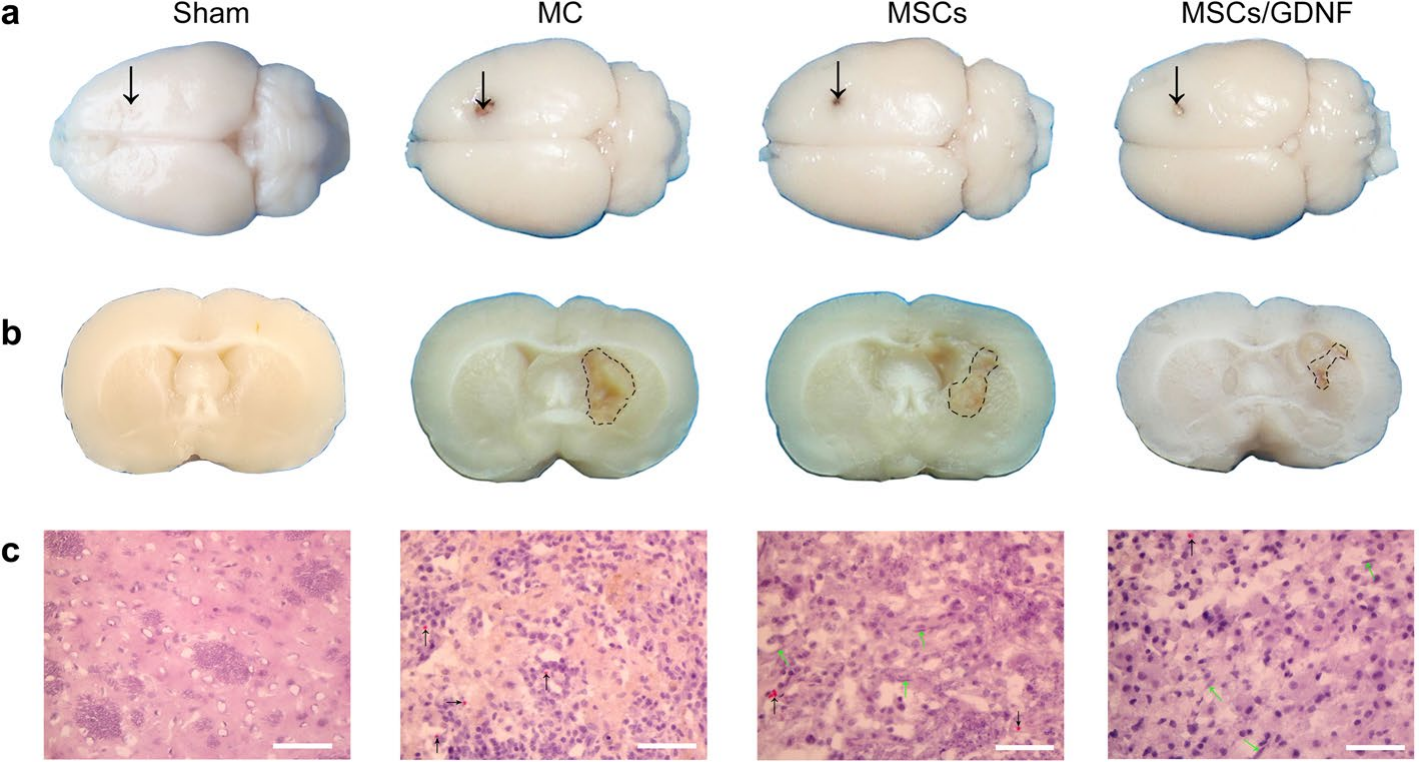
MSCs/GDNF treatment improves brain tissue structure. **a** The upper views of whole rat brains in different groups. The arrow represents the needle entry point. **b** Coronal sections of rat brains show the hemorrhagic focus in different groups. The dashed line area indicates the hemorrhagic focus. **c** H&E staining shows pathological structure of brain tissues of rats in different groups. Black arrow: red blood cell; green arrow: transplanted cell. Scale bar, 100 μm

After ICH, the size and severity of the lesion are directly related to the degree of neurological impairment. Here, we observed the site and histopathological changes of hemorrhagic focus. The coronal sections of the brains showed that hemorrhage foci were visible in MC, MSCs and MSCs/GDNF groups, and the lesion areas were located mainly in the striatum. The MSCs/GDNF group displayed a smaller lesion area than the MC and MSCs groups (Fig. 3a-b). H&E staining of the brain tissues indicated that the brain tissue surrounding the hematoma was loose, and a small amount of red blood cells were seen. Furthermore, some implanted cells were in the focal area, and MSC and MSCs/GDNF transplantation improved histological structure (Fig. 3c).

**Fig. 4:**
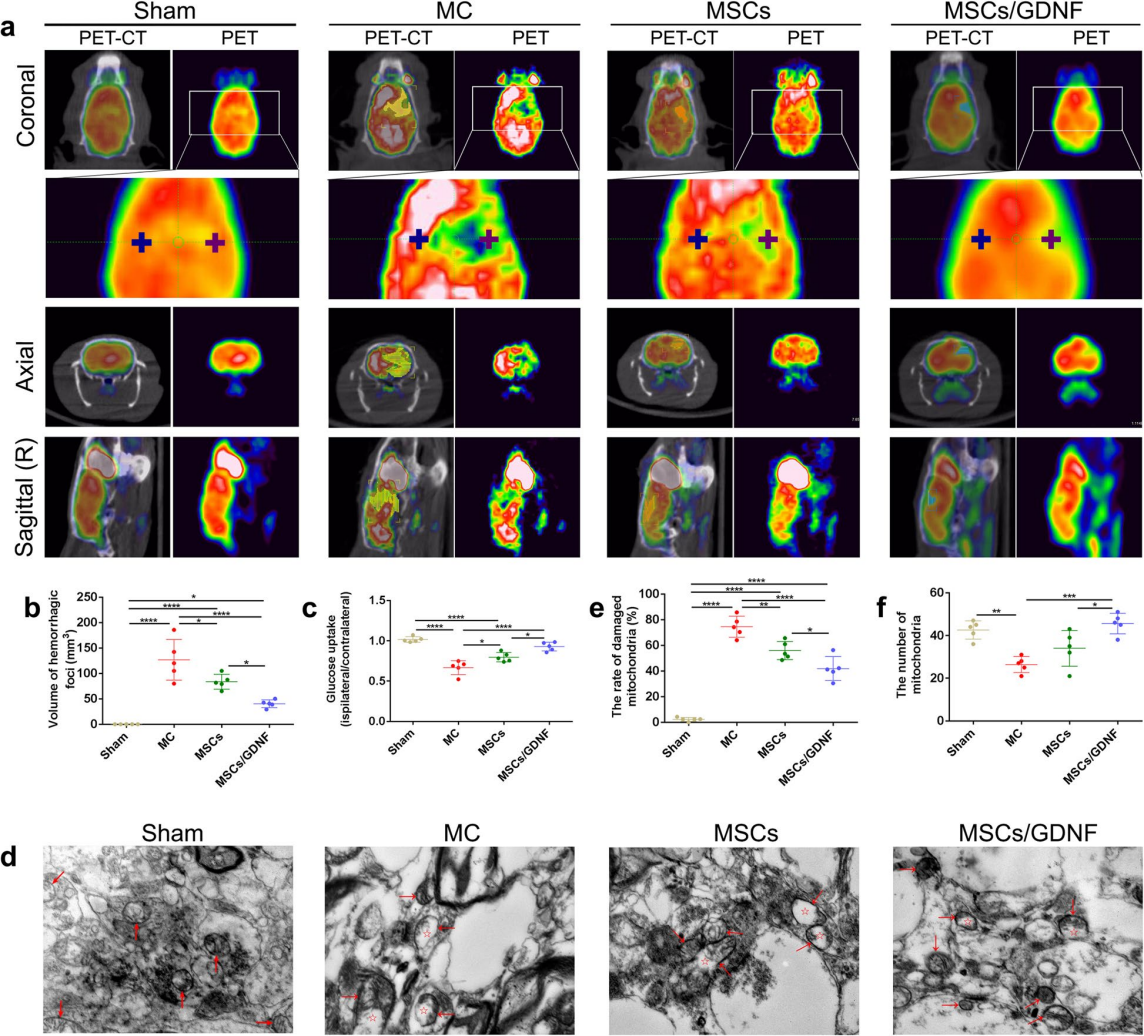
MSCs/GDNF treatment improves glucose uptake level and mitochondrial structure. **a** PET-CT images of rat brains in the different groups. Pluses indicate the points where glucose uptake was measured, and the shadow represents the hemorrhagic focus. **b** The quantitative analysis of lesion volume via ^18^F-FDG micro-PET-CT scan (*n* = 5 per group). **c** The quantitative analysis of glucose uptake (ipsilateral/contralateral) via.^18^F-FDG micro-PET-CT scan (*n* = 5 per group). **d** Representative electron micrographs of the peripheral area of the bleeding foci in the different groups. Red arrow: mitochondria; red pentagram: part of the mitochondrial vacuole. **e** The percentage of damaged mitochondria in the different groups (*n* = 5 per group). **f** The number of mitochondria in the different groups (*n* = 5 per group). Scale bar, 1 µm. *P < 0.05, **P < 0.01 and ****P < 0.001

In exploring a better evaluation of the effect of cell transplantation on ICH, we detected the volume and glycometabolism level of the hemorrhagic focus in different groups by PET/CT. The PET/CT scan showed no bleeding lesion in the Sham group, but the bleeding foci were evident in other groups. Moreover, the two cell transplantation groups displayed reduced volumes of the lesion areas and elevated the glucose uptake (ipsilateral/contralateral) compared with the MC group, and the MSCs/GDNF group showed a better outcome than the MSCs group (*p* < 0.05, Fig. 4a-c). We next performed electron microscopy to further verify whether cell transplantation altered the number and ultrastructure of mitochondria in the ICH brain. As shown in Fig. 4d-f, the mitochondrial crista was transparent, and the mitochondrial membrane was intact in the sham group. Still, the mitochondria appeared crista fracture and vacuole in the MC group. Cell transplantation significantly reduced the percentage of damaged mitochondria (*p* < 0.05). Importantly, the MSCs/GDNF group increased the number of mitochondria and decreased remarkably the rate of damaged mitochondria compared with the MSCs group (*p* < 0.05). Taken together, our results suggest that GDNF produced by transplanted MSCs/GDNF could reduce lesion size by improving mitochondrial quality and function.

### MSCs/GDNF transplantation suppresses neuroinflammation

**Fig. 5:**
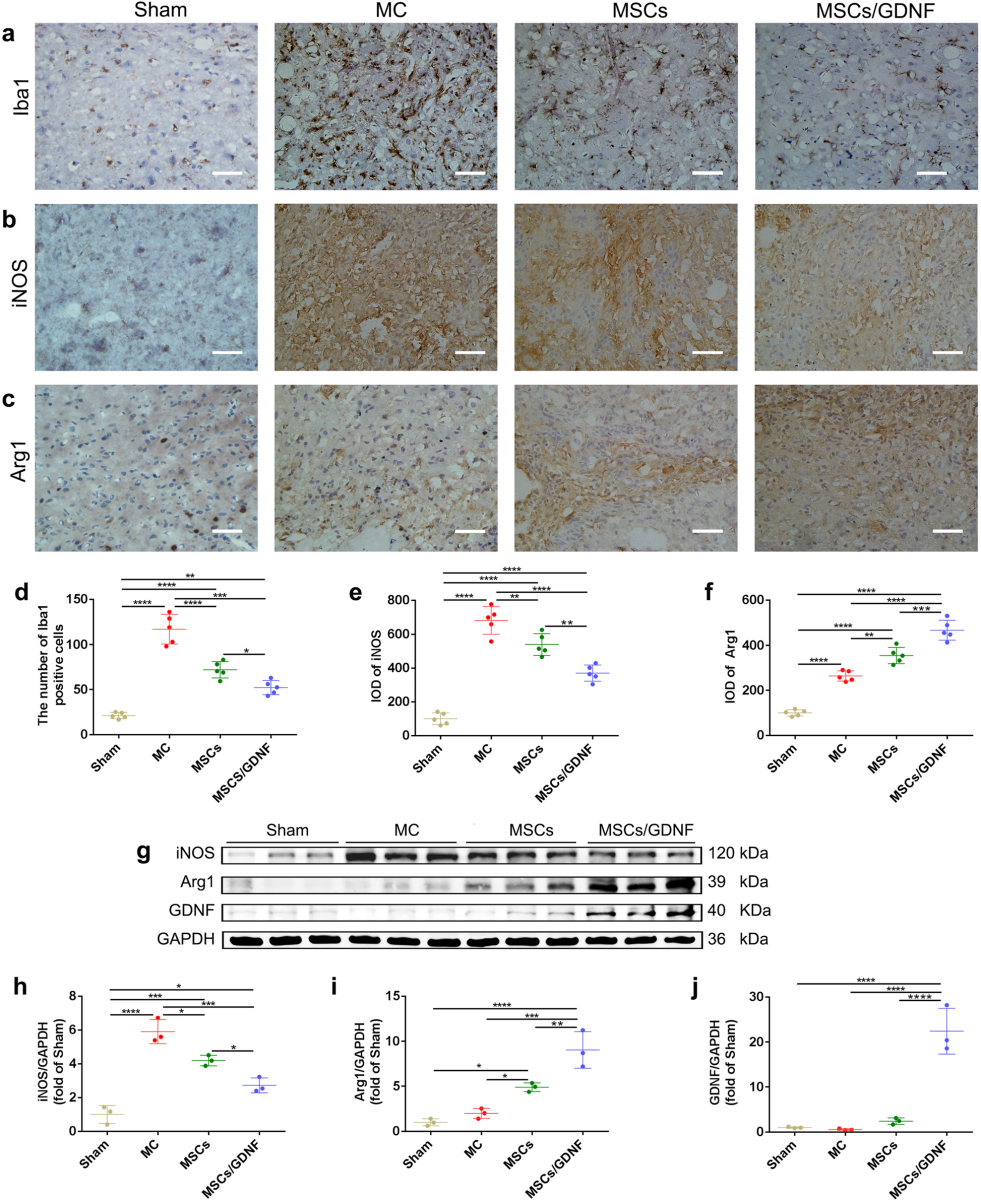
The effect of MSCs/GDNF transplantation on microglia. **a-c** The images of Iba1, iNOS, Arg1 IHC staining in the ipsilateral striata of rats. **d-f** Quantitative analysis of the expressions of Iba1, iNOS, and Arg1 immunopositive staining. **g** Arg1, iNOS and GDNF protein bands were determined by Western blotting. **h-j** Quantitative analyses of Western blot bands showing GDNF, iNOS and Arg1 protein levels in different groups (*n* = 3 per group). Scale bars, 100 μm (**a-c**). *P < 0.05, **P < 0.01, ***P < 0.001, and ****P < 0.0001

ICH activates microglia and causes neuroinflammation. Here the expressions of iNOS and Arg1 proteins were determined to assess the degree of inflammatory response. Immunohistochemistry (IHC) staining revealed that compared with the Sham group, the number of ionized calcium binding adapter protein-1 (Iba1, one of the microglia markers) -positive cells increased rapidly in the MC group, but the MSCs and MSCs/GDNF transplantation reversed notably the changes in the ICH rats (*P* < 0.05, Fig. 5a, d). Moreover, the MSCs/GDNF group reduced obviously the number of Iba1-positive cells compared to the MSCs group (*P* < 0.05, Fig. 5a, d). Microglia in the brain is polarized to pro-inflammatory (M1) and anti-inflammatory (M2) phenotypes after ICH. The results of the IHC staining and Western blotting indicated that the levels of inducible nitric oxide synthase (iNOS, M1 marker) and arginase-1 (Arg1, M2 marker) proteins in the MC group were increased significantly when compared with that in Sham group, and both MSCs and MSCs/GDNF transplantation downregulated iNOS protein but upregulated Arg1 protein in ICH rats (*P* < 0.05, Fig. 5b-c, e-i). Furthermore, the MSCs/GDNF transplantation downregulated the iNOS protein expression but upregulated the Arg1 protein expression compared with MSC transplantation (*P* < 0.05, Fig. 5b-c, e-i). In addition, the GDNF expression level in the ipsilateral striatum in the MSCs/GDNF group was significantly higher than in the other groups (*P* < 0.05, Fig. 5g, j). Our results suggest that GDNF produced by MSCs/GDNF can alleviate inflammatory response.

### MSCs/GDNF transplantation promotes angiogenesis

**Fig. 6:**
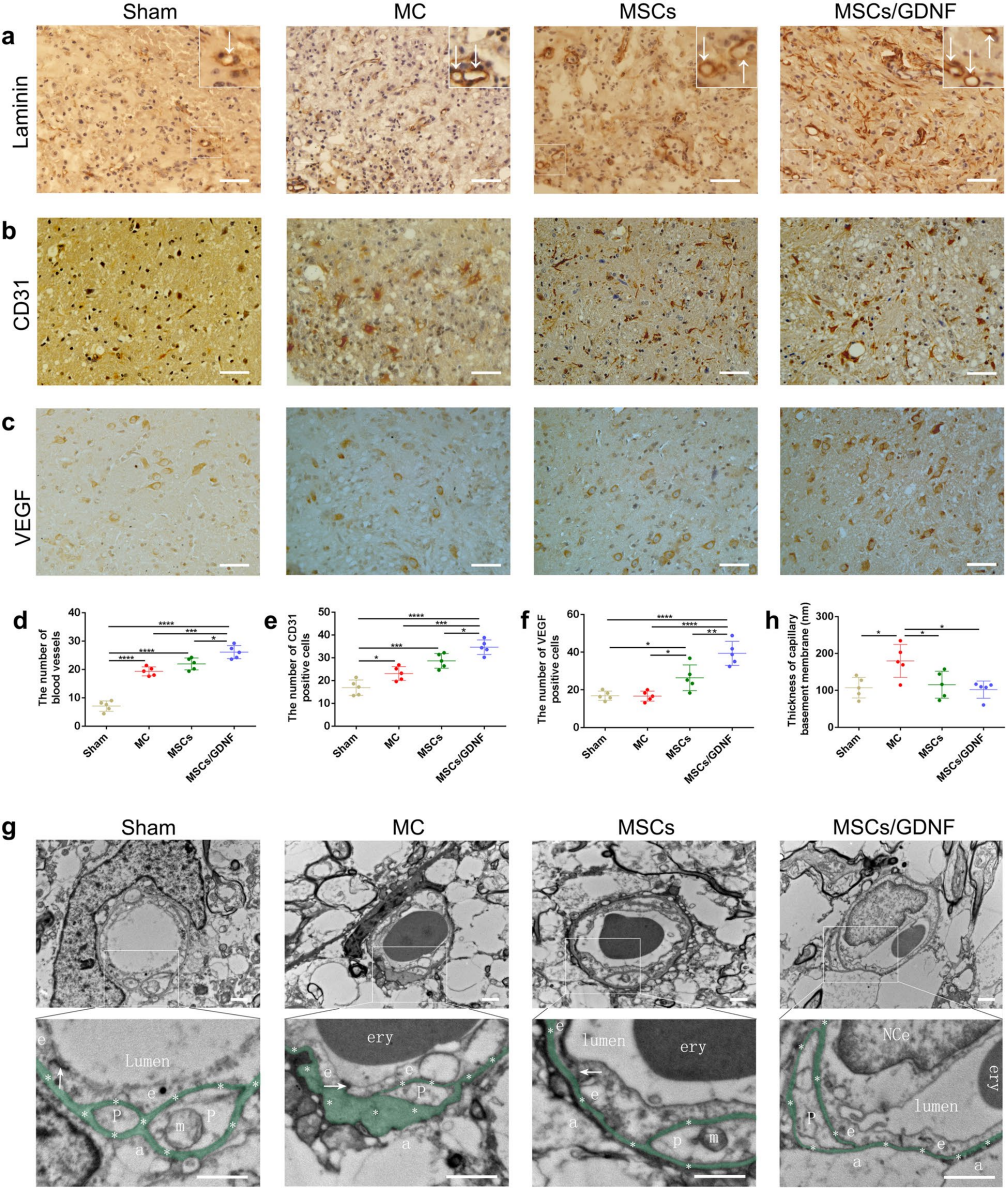
The effect of MSCs/GDNF transplantation on blood vessels and associated proteins. **a**-**c** The images of laminin, CD31, VEGF IHC staining in ipsilateral striata of rats. **d**-**f** The numbers of blood vessels, CD31- and VEGF-positive cells in the striata of the different groups (*n* = 5 per group). **g** Representative electron micrographs of brain capillaries in the different groups. The arrowhead indicates a tight junction between two endothelial cells. Asterisks show the basement membrane of the brain capillary. NCe, the nucleus of the endothelial cell; ery, erythrocyte; e, endothelial cell; p, pericyte; a, foot of astrocyte; m, mitochondrion. **h** Quantitative analysis of the thickness of capillary basement membrane. Scale bar, 100 μm (**a-c**) and 1 μm (**g**). *P < 0.05, ***P < 0.001, and ****P < 0.0001

To investigate possible mechanisms underlying the effect of MSCs/GDNF transplantation on angiogenesis, we detected the expression of essential proteins related to blood vessels. IHC staining of laminin revealed that the number of blood vessels in the MC group was more than that in the Sham group, and that markedly increased in the MSCs/GDNF group when compared to the other groups (*P* < 0.05, Fig. 6a, d). Furthermore, the expressions of vascular-related proteins (CD31 and VEGF) were also detected by IHC staining. The experimental outcome indicated that cell transplantation significantly improved the amounts of CD31- and VEGF-positive cells in the ICH rats, and the MSCs/GDNF transplantation increased further the number of the two positive cells compared to MSC transplantation (*P* < 0.05, Fig. 6b-c, e–f). Transmission electron microscopy (TEM) showed that the basement membrane of capillaries was thickened locally in the MC group. Still cell transplantation thinned the thickness of capillary basement membrane in ICH rats (*P* < 0.05, Fig. 6g-h). In addition, the capillary ultrastructure of each group was intact (Fig. 6g). In general, the results show that MSCs/GDNF transplantation facilitates angiogenesis.

### MSCs/GDNF transplantation alleviates brain injury

**Fig. 7:**
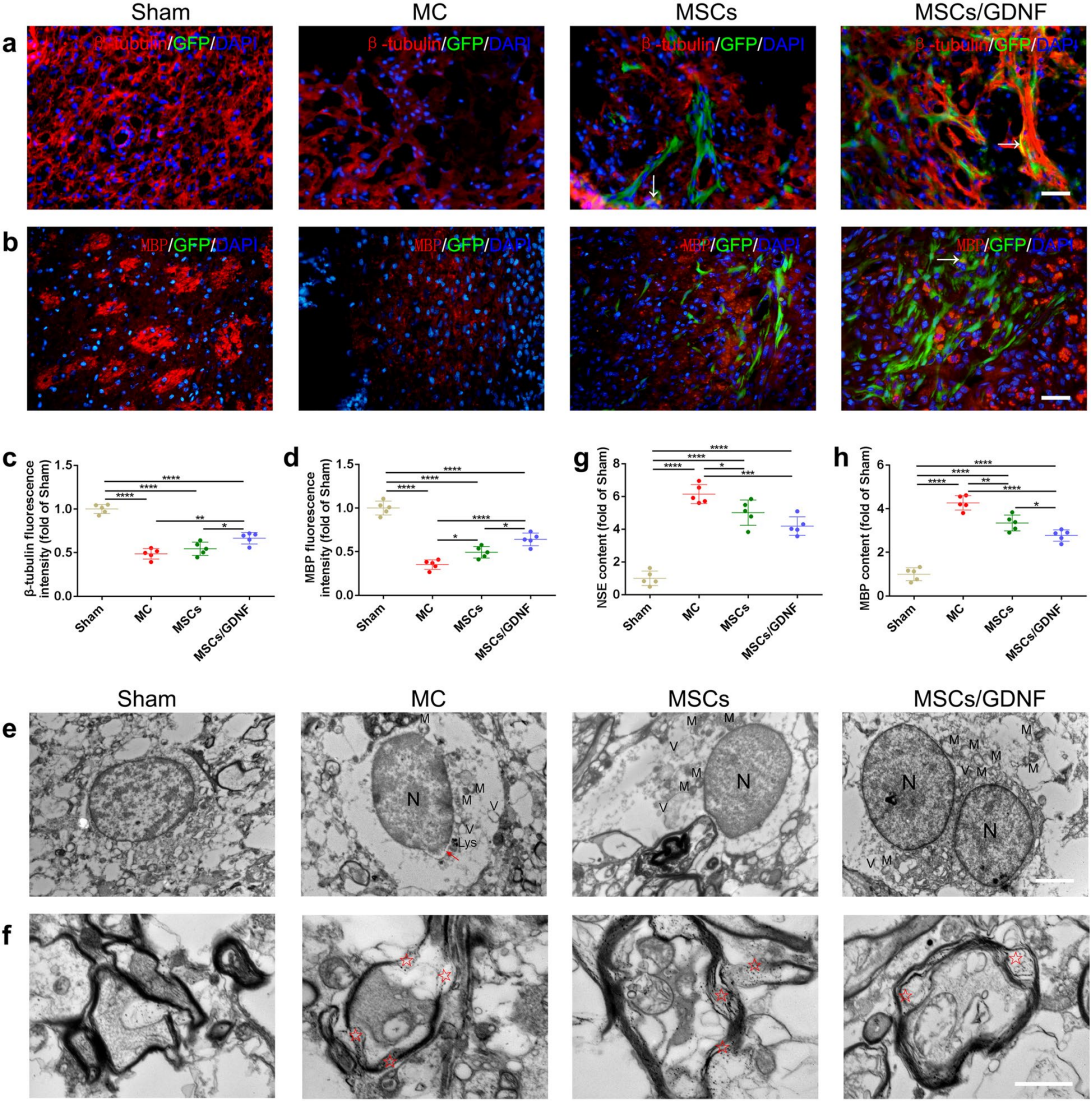
The effect of MSCs/GDNF transplantation on neurons and the myelin sheath. **a**-**b** The images of β-tubulin and MBP IF staining in different groups. White arrows indicate the transplanted cells expressing β-tubulin or MBP protein. **c**-**d** Relative fluorescence intensities of β-tubulin and MBP (*n* = 5 per group). **e**–**f** Electron micrographs of neurons (**e**) and the myelin sheath (**f**) in the striata of the different groups. Red arrows show defective or blurred nuclear membranes, and red pentagrams represent demyelination. N, nucleus; V, vacuole; M, mitochondrion; Lys, lysosome. (**g**-**h**) ELISA shows the NSE content (**g**) and MBP content (**h**) in sera of the different groups (*n* = 5 per group). Scale bars, 100 μm (**a-b**), 2 μm (**e**) and 1 μm (**f**). *P < 0.05, **P < 0.001, and ****P < 0.0001

We analyzed the changes of neurons and myelin to further clarify whether MSCs/GDNF transplantation lessen brain damage. As shown in Fig. 7a-b, immunofluorescence staining indicated that ICH resulted in a significant decrease in the fluorescence intensity of β-tubulin, a neuronal marker and MBP, an oligodendrocyte marker. The MSCs/GDNF transplantation enhanced the fluorescence intensity of β-tubulin, and both MSC and MSCs/GDNF transplantation significantly improved the fluorescence intensity of MBP in the rats with ICH (*P* < 0.05, Fig. 7a-d). In the two cell transplantation groups, the fluorescence intensities of β-tubulin and MBP were higher in the MSCs/GDNF group (*P* < 0.05, Fig. 7a-d). Electron microscopy showed neuronal edema and demyelination in MC, MSCs and MSCs/GDNF groups, with more severe ultrastructural damage in the MC group (Fig. 7e-f). In addition, ELISA experiments revealed that the NSE and MBP concentrations decreased markedly in ICH rats after cell transplantation, and MSCs/GDNF transplantation could further reduce the MBP concentration compared with MSC transplantation (*P* < 0.05, Fig. 7g-h). The results were consistent with those observed by immunofluorescence and TEM. Overall, the data demonstrate that MSCs/GDNF transplantation can protect brain tissue in ICH better than MSCs transplantation.

### MSCs/GDNF transplantation enhances synaptic plasticity

**Fig. 8:**
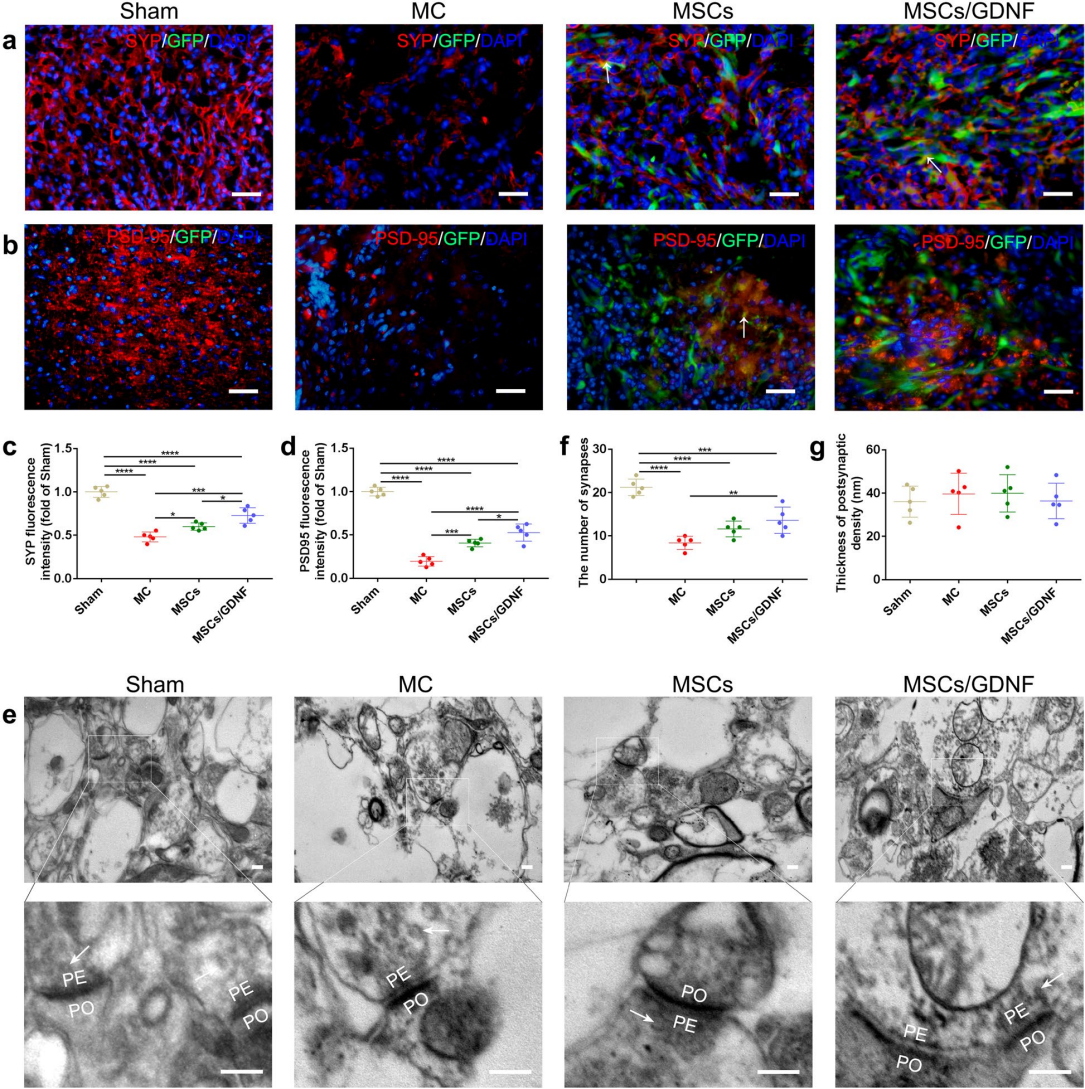
The effect of MSCs/GDNF transplantation on synaptic plasticity. **a**-**b** The images of SYP and PSD-95 IF staining in the different groups. White arrows indicate the transplanted cells expressing SYP or PSD-95 protein. **c**-**d** Relative fluorescence intensities of SYP and PSD-95 (*n* = 5 per group). **e** The electron micrographs of synapses around hemorrhagic foci. The white arrows show synaptic vesicles. PO, postsynaptic element; PE, presynaptic element. **f**-**g** The number of synapses and thickness of postsynaptic density in the different groups (*n* = 5 per group). Scale bars, 100 μm (**a-b**) and 200 nm (**e**). *P < 0.05, **P < 0.01, ***P < 0.001, and ****P < 0.0001

To determine whether cell transplantation had an impact on synaptic plasticity in the post-hemorrhagic brains of rats, we performed immunofluorescence staining for synaptophysin (SYP) and postsynaptic density protein 95 (PSD-95) one week after cell transplantation. The results showed that the levels of SYP and PSD-95 in the MC group were significantly lower than in the Sham group. Cell transplantations notably increased the expressional levels of SYP and PSD-95 proteins, with the MSCs/GDNF transplantation being superior to the MSC transplantation (*P* < 0.05, Fig. 8a-d). In addition, a few grafted cells labeled by GFP protein co-expressed SYP and PSD-95. Meanwhile, TEM showed that a typical synapse in each group had distinct presynaptic and postsynaptic endings separated by a synaptic cleft, and many synaptic vesicles were located in the presynaptic end, but the postsynaptic end had a thicker membrane (Fig. 8e). ICH resulted in the destruction of synapses and the peripheral ultrastructure, which was partially rescued by cell transplantation. In terms of thickness of postsynaptic density, there was no statistical difference among all groups, although the MSCs/GDNF transplantation improved the number of synapses in the ICH rats (*P* < 0.05, Fig. 8e-g). Our experiments show that MSCs/GDNF transplantation helps enhance synaptic plasticity.

## Discussion

Among the leading causes of death worldwide, stroke ranks second, and incidence in developing countries is rising. ICH accounts for 10–20% of all strokes [21]. Brain injury after ICH involves a series of pathological processes: blood–brain barrier (BBB) breakdown, brain edema, neuroinflammation, oxidative stress, iron deposition, and cell apoptosis. These changes are associated with poor neurological prognosis and high mortality [22–24]. Animal experiments and clinical trials have shown that MSC transplantation can improve the prognosis after ICH, but its efficacy needs to be further strengthened [6, 25]. GDNF is a neurotrophic factor that regulates motor neuron survival and neurite outgrowth [26]. However, its clinical application is limited because of its difficulty crossing the BBB, short half-life, and rapid degradation rate [27]. Here, we transduced the GDNF gene into MSCs and transplanted the cells into the focal area of ICH rats to bypass the BBB and continuously generate GDNF and investigated the changes in brain structure and gait, as well as neuroinflammation, angiogenesis, cell survival, and synaptic plasticity.

In this experiment, animal gait data were adopted to compare the neurological function of the model and cell transplant therapeutic ICH rats, as reported by Fan and coworkers [28]. For a more comprehensive assessment of gait changes, 30 indicators were selected in this study. We found that ICH caused noticeable changes in mostly measured gait parameters. Furthermore, our data revealed significant differences between the MC group and cell transplantation groups in some gait parameters, including the regularity index of normal step sequence, run speed, print area, track width, stride length, stride time, homolateral coupling, diagonal coupling, stance time, and swing time. The data suggested that cell transplantation could significantly improve the gait function in rats after ICH. More significantly, in terms of the core gait parameters that reflect the rats' overall motor ability (such as the rate of normal step sequence, run speed, and limb coordination), the therapeutic effect of MSCs/GDNF transplantation was better than that of MSC transplantation. In addition, we found 10 gait parameters that did not differ significantly among all groups, and eight of them belong to forelimbs. On one hand, the results revealed that ICH and cell transplantation couldn’t cause apparent changes in these parameters at this time-point. On the other hand, ICH and cell transplantation had a lesser effect on the forelimbs than on the hind limbs at this time point, which may be related to the roles of the forelimbs and hind limbs in walking.

During a hemorrhagic event, the continuous supply of glucose and oxygen to the cells in the focal area is seriously impaired, which results in decreased cellular function in the lesion and peripheral area. ^18^F-FDG is a glucose analogue and has been adopt to examine the brain’s glucose consumption [29]. Furthermore, ^18^F-FDG PET is non-invasive and is often used to measure the impacts of various therapeutic strategies on stroke without disrupting physiology [30, 31]. Feng, et al. reported that MSC transplantation could increase the ^18^F-FDG accruement at the ipsilateral basal ganglia in *Macaca fascicularis* monkeys after ICH [32]. In the present study, using ^18^F-FDG PET/CT, we found that transplantation of MSC overexpressing GDNF significantly enhanced the effect of cell therapy in the rats with ICH. Brain cells are susceptible to energy variation and need to maintain the stability of mitochondrial structure and function to provide sufficient energy, since mitochondria are the primary producers of the ATP required for the normal electrical activities of cells; therefore, mitochondrial damage can lead to cell death [33, 34]. Our experiment showed that MSC implantation reduced the percentage of damaged mitochondria. Interestingly, MSCs/GDNF transplantation improved the number and ultrastructure of the mitochondria compared with the MSC transplantation. These data suggested that the GDNF secreted by MSCs/GDNF helps maintain mitochondrial stability and promotes cell survival.

Microglia, the resident immunocytes in the brain, can be expeditiously activated after ICH and play a critical role in mediating the neuroinflammatory reaction [35, 36]. Activated microglia around the hematoma not only induce an acute inflammatory reaction, excitotoxicity, oxidative stress, and cytotoxicity to result in brain cell death (M1 phenotype), but also can convert to the opposite M2 phenotype that eliminates the hematoma, suppress inflammation, and facilitate tissue regeneration [37]. Studies have shown that changing the microglial phenotype from M1 to M2 had a neuroprotective effect in ICH animals [38, 39]. Previous experiments indicated that MSC transplantation attenuated microglial activation, the levels of pro-inflammatory cytokines and neutrophil infiltration at the ICH site [40, 41]. In this experiment, ICH led to increased expression of Iba1, iNOS and Arg1 proteins. Cell transplantations reduced the number of Iba1-positive cells and decreased iNOS expression, but increased Arg1 expression. These data suggest that MSC transplantation and MSCs/GDNF transplantation can inhibit the over-activation of microglia and promote the transformation of microglia from the M1 phenotype to the M2 phenotype. Most importantly, transplantation of MSC overexpressing GDNF enhanced the anti-inflammatory effect compared with single cell transplantation in ICH rats.

After ICH, the hematoma formed has a space-occupying effect and rapidly destroys regional blood flow, thus leading to an ischemic effect on the brain tissue near the hemorrhage [42]. Some studies have shown that neovascularization in the tissues adjacent to the hematoma is stimulated, which may be a positive repair process for the recovery of neurological function [43, 44]. Upregulation of many molecules, including laminin, CD31, and VEGF, is involved in this process. Laminin, one of the extracellular matrix proteins in vascular basement membrane, has been used to observe and count blood vessels via immunohistochemical staining [45, 46]. CD31 is an endothelial marker and often utilized to evaluate angiogenesis [47]. Numerous studies have shown that VEGF can stimulate capillary repair [48, 49]. VEGF is a highly bioactive dimer glycoprotein that acts explicitly on vascular endothelial cells to regulate proliferation, migration, and lumen formation and is also the critical factor in the formation of the collateral circulation that bypasses the blocked vasculum in the disease in adult organisms [43]. In the present study, ICH promoted vascular proliferation, and the MSCs/GDNF treatment increased the number of blood vessels relative to saline injection and MSC treatment, but the ultrastructure of capillaries in all four groups was intact. After treatment with cell transplantation, there was an increase in VEGF- and CD31-positive cells around the perihemorrhagic zone, and the effect of MSCs/GDNF transplantation is more significant. These data suggest that the GDNF secreted by GDNF/MSCs could contribute to angiogenesis.

Cell death, which is closely related to the microenvironment, is an important prognostic factor of ICH that can predict the pathological structure of brain tissue and poor functional outcomes [50, 51]. GDNF, a member of the TGF-β superfamily, contributes to improving the local microenvironment and promoting cell survival, reinnervation, and neural function recovery [19, 52–54]. However, the application of GDNF in the effective treatment of diseases is limited since the BBB restrains the local delivery of macromolecular therapeutic agents into the CNS [55]. Therefore, GDNF gene therapy is an ideal strategy for its clinical application. Our previous experiments showed that GDNF/MSCs expressed GDNF in vitro and rats’ brains after ICH. The MSCs/GDNF transplantation reduced neurological dysfunction, shrank the lesion area, and inhibited cell apoptosis in ICH rats compared to the MSC transplantation [19]. In this experiment, we found that the transplantation of GDNF-overexpressing MSCs could improve β-tubulin as well as MBP protein expressions in ICH rats compared with the transplantation of MSCs, but only a few of the transplanted cells expressed β-tubulin and MBP proteins in both MSCs and MSCs/GDNF groups. These results suggested that cell transplantation mainly promotes the survival of host endogenous neurons and oligodendrocytes, and the MSCs/GDNF transplantation was more effective.

ICH causes the injury and death of neurons, leading to the damage of synapse structures and a reduced number of synapses, which is critical for neurological function [56, 57]. The interventions of exercise, Mdivi-1, and electro-acupuncture stimulation increased the synapse number and the level of synaptic plasticity-associated proteins (e.g. PSD-95 and SYP) in the rat brain after ICH [57, 58]. PSD-95 is a central part of the postsynaptic terminal of glutamatergic excitatory synapses and plays essential roles in physiology and behavior [59]. SYP, an integral membrane glycoprotein of synaptic vesicles, has been established as a marker for synaptic density and synaptogenesis [60]. MSCs have been shown to be an influential factor in reversing cognitive aging and neurological function via synaptic plasticity [61, 62]. Our study found that the MSCs/GDNF intervention increased the expression of PSD-95 and SYP proteins compared with the MSC intervention and improved the number of synapses compared with no intervention in ICH rats. The data revealed that the MSCs/GDNF transplantation improved synaptic plasticity.

Although various methods including gene therapy and drug conveying systems have been developed to supply growth factors to locally damaged nerves, finding a suitable way to provide GDNF to promote nerve repairment is still a great challenge [63]. Overexpression of GDNF or NGF in transgenic mice’s skin could alter the transcriptional plasticity of nociceptive sensory neurons caused by inflammation [64]. Overexpression of GDNF mediated by lentiviral vector in peripheral nerve resulted in supra-physiological levels of GDNF accumulation, which could cause trapping of regenerating axons, injuring both long-distance growth of motoneuronal axons and reinnervation [65, 66]. This ‘trapping of regenerating axons’ might be triggered by the relatively high level (100-fold) in GDNF expression, since the effect caused by GDNF seemed to be dose dependent [66, 67]. In this experiment, the transplantation of MSCs/GDNF resulted in an approximately 20-fold increase in GDNF levels compared with the Sham group, and the side effects caused by overexpression of GDNF have not been found according to the indicators tested so far.

There are some deficiencies in this study. First, we only observed the relevant indicators at 7 days after cell transplantation, and the long-term effects of MSCs/GDNF transplantation on ICH remain unclear. Second, the engraftment efficiency of the MSCs and the dose of transplanted cells to achieve the best outcome were not investigated. In addition, although the morphological changes of capillary walls, medulla, synapses and other structures were observed, there is a lack of sufficient quantitative data and studies on the relevant influencing mechanisms.

**Fig. 9:**
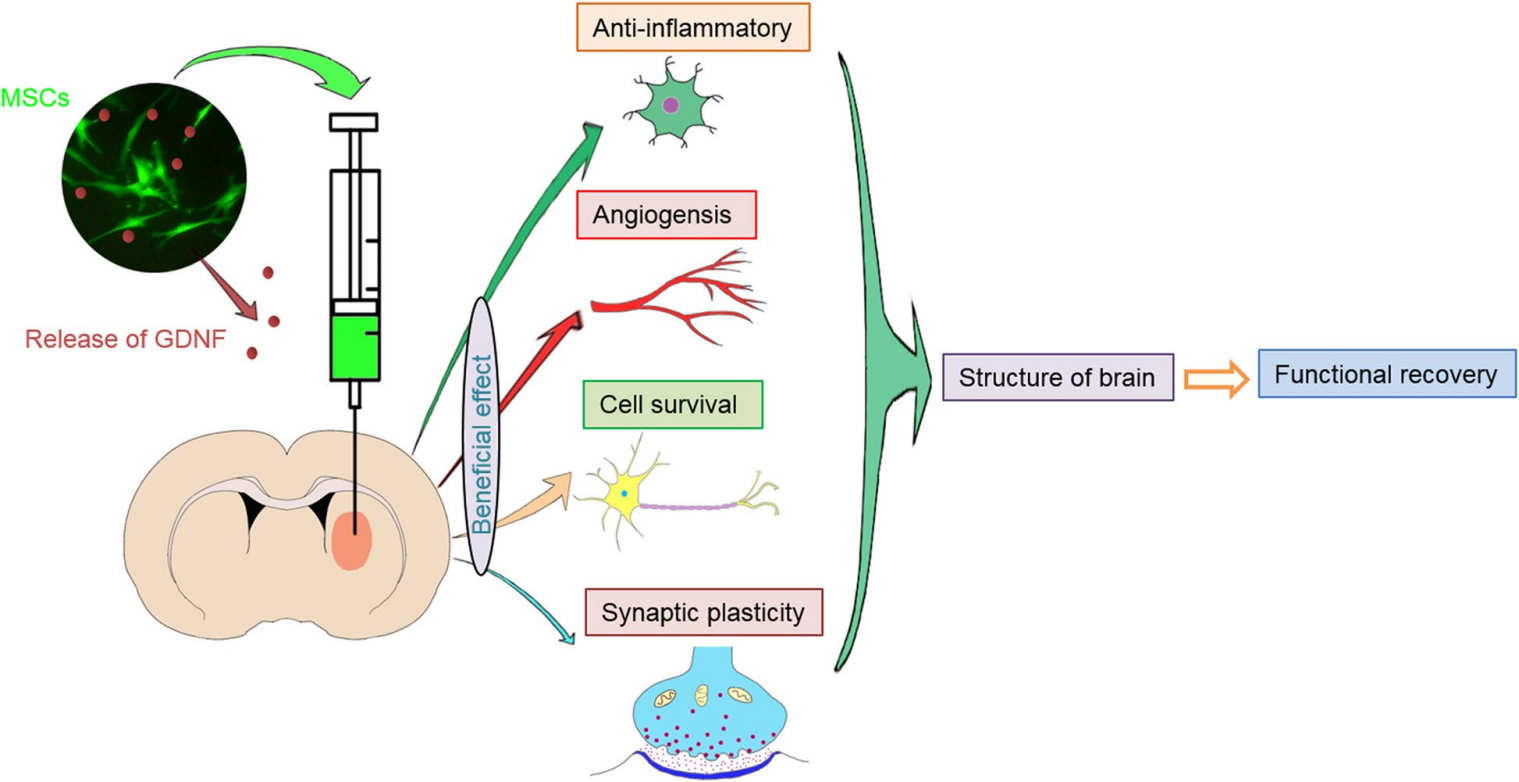
Graphical summary

In summary, although this study has some limitations, the current study demonstrated that MSC transplantation and MSCs/GDNF transplantation generate neuroprotective effects in ICH rats. More importantly, our experiments proved that the transplantation of GDNF-overexpressing MSCs achieved better results by facilitating the survival of oligodendrocytes and neurons, offering beneficial effects on angiogenesis, inhibiting inflammation, and enhancing synaptic plasticity. These changes could improve brain structure and neurobehavioral function in rats after ICH (Fig. 9). Therefore, based on the present study and our previous investigations we put forward a strategy of transplanting MSCs overexpressing GDNF to treat nervous system diseases such as stroke, Parkinson's, and Alzheimer's. However, further investigations on the drawbacks mentioned in this study are required. It is imperative in preclinical studies to address the potential clinical impediments such as off-target effects of GDNF overexpression, high levels of GDNF gene expression and their possible side effects.

## Materials and methods

### Experimental animals

The male SD rats used for this study were offered by the Experimental Animal Center of Southwest Medical University (Luzhou, China). The rats were raised at an appropriate temperature (23 ± 2 °C) with 12 h of dark/light circulation, free drinking and eating. The rat experimental protocols were approved by the Animal Care and Use Committee at the Southwest Medical University.

### MSC culture and infection with adenovirus

MSCs were obtained and cultivated as mentioned in our previous experiments described [18]. Simply put, after anaesthetizing SD rats (weighing 100–120 g) with pentobarbital sodium, the femurs and tibias were isolated and the epiphyses were cut off, then bone marrow was collected by douching the medullary cavity with Dulbecco’s Modified Eagle Medium (DMEM)-low glucose (L-DMEM) (Gibco). MSCs were purified from the bone marrow using Percoll density gradient medium (Solarbio) and cultured in L-DMEM supplemented with 10% fetal bovine serum (FBS) (Hyclone), penicillin (1 × 10^5^ U/L) and streptomycin (100 mg/L) (Beyotime) in a CO_2_ incubator (5% CO_2_, 37 °C). When the cells reached 80% ~ 90% confluence, they were digested with the Trypsin–EDTA Solution (Beyotime), re-suspended in a medium, and then subcultured.

The MSCs were infected with adenovirus as previously reported [19]. Both GDNF gene recombinant adenovirus (pAdEasy-1-pAdTrack-CMV-GDNF) and control adenovirus (pAdEasy-1-pAdTrack-CMV) were diluted to 4 × 10^6^ PFU/ml in phosphate-buffered saline (PBS). The fourth passage MSCs were seeded in 25 cm^2^- culture flasks at a 1.5 × 10^4^/cm^2^ density. When the cells grew up to 70% ~ 80% confluence, 1.5 ml GDNF or control adenovirus solution was put in different culture flasks, then the culture flasks were shaken slowly from time to time and the cells were incubated for 3 h. After that, the cells were cultured with medium for 48 h to achieve MSCs/GDNF and MSCs (control). The cells were collected and the cell concentration was adjusted to 2.5 × 10^7^/ml in saline for follow-up experiments.

### Experimental groups and rat ICH model

Adult SD rats (weighing 220–250 g) were assigned randomly to 4 experimental groups (*n* = 13 in each group): the Sham operation (Sham) group; the model control (MC) group containing ICH-induced animals that received 20 μL saline; the MSC transplantation (MSCs) group containing ICH-induced animals that received 5 × 10^5^ MSCs in 20 μL saline; and the MSCs/GDNF transplantation (MSCs/GDNF) group containing ICH-induced animals that received 5 × 10^5^ MSCs/GDNF in 20 μL saline.

The method for establishing rat ICH model is as follows: After anaesthetization with 2% pentobarbital sodium (40 mg/kg), the rat was placed on a stereotaxic frame (Benchmark), H_2_O_2_ was used to expose the anterior fontanel, and a burr hole was drilled into the right cranial bones; the solution containing 2 μL collagenase type I (Sigma) (0.125 U/µL) and 1 μL heparin (Sigma) (2 U/µL) was injected into the striatum (location: 0.2 mm anterior, 3 mm right, and 6.5 mm ventral to the anterior fontanel) via a microinjector at 0.3 μL per min; then, the needle was slowly removed. Rats in the sham group underwent the same procedure as described above, except for injections of collagenase and heparin.

### Cell implantation

Two days following ICH induction, the rats were anaesthetized and put on a stereotaxic frame once more. Then, a sterile microsyringe was used to absorb 20 μL saline or cell suspension (MSCs or MSCs/GDNF, 5 × 10^5^ cells in saline) and fixed on the stereotaxic frame. The needle was inserted into the right striatum through the drill hole in the skull as described above (location: 6 mm vertical deep from the burr hole). Saline or cell suspension was injected at 2 μL per minute. After the injection was completed, the needle was stopped for 5 min, and then slowly withdrawn. The creatures survived for 1 week after injection of saline or cell implantation.

### Gait analysis

The neurological functions of the rats were evaluated with the TreadScan Gait System (Clever System Inc, USA). The equipment, which mainly consists of a high-speed digital video recorder and a treadmill device, was used to determine animal’s gait following a protocol. For each trial, the rat was put on the treadmill for 20 s, running at a speed of 8 cm/s, and a video of their footprints was recorded at 100 frames per second. The captured frames were analyzed using TreadScan software by an individual who was blinded to the gait parameters and outcomes to assess gait performance. This experiment’s measured parameters included regularity index of normal step sequence, run speed, print area, track width, swing time, stance time, stride length, stride time, homolateral coupling, and diagonal coupling. Coupling parameters were used to measure the degree of pace consistency, which was obtained by dividing the lag landing time of the target foot relative to that of the reference foot by the current gait occurrence time of the reference foot (from this landing to the next landing). For homolateral coupling, the closer the value to 0.5, the more coordinated the pace. For diagonal coupling, the closer the value to 0, the more consistent the pace.

### PET/CT

Micro-PET/CT (Siemens, Germany) was used to obtain positron emission tomography/computed tomography (PET/CT) imaging of experimental rat brains according to previous methods [68]. After injection with ^18^F-fluorodeoxyglucose (^18^F-FDG) in the tail vein (dose: 0.8 mCi/kg), the rat was placed in its cages to permit the ^18^F-FDG ingestion for 30 min. Then, the animal was put in the micro-PET/CT scanner under narcotization, and PET scan as well as CT scan, was executed on the head. The PET/CT imaging of the rat was analyzed with ASIPro VM software and PMOD software to detect the glucose metabolism level and lesion volume, and the assessment was finished by one blind operator. The volume of the lesion was calculated as follows: PET/CT fusion images showing sparse distribution or absence of imaging agents were positive (the uptake ratio between the ipsilateral side and the contralateral side was less than 0.5), and the reduced glucose uptake or the margin of the defect area was delineated at each layer according to the size of the hematoma, then the volume of hematoma in the different groups was calculated using a micro-PET/CT semi-automatic volume measurement tool.

### Pathology examination

Five rats in each group were anesthetized One week after treatment through intraperitoneal injection of excessive stupefacient, then transcardially perfused with ice-cold physiological saline and 4% paraformaldehyde (PFA) successively. The brains removed from the skull cavities, divided into anterior and posterior parts, post-fixed overnight with 4% PFA at 4 ^◦^C, and subsequently dehydrated with 30% sucrose and embedded with O.C.T. compound (Biosharp). After that, we cut the brain tissues into 7 µm thick coronal slices with a cryotome (Leica) and conserved them in a fridge at -18 °C until used for histological staining. The slices were dyed in hematoxylin and eosin solution for pathology estimate.

### Immunohistochemistry (IHC) and immunofluorescence (IF)

For IHC, the cryosections were sequentially treated as follows: washed with 0.01 M phosphate buffered saline (PBS) 3 times for 5 min each time at room temperature (RT), immersed in 0.3% Triton X-100 for 20 min at RT, washed with PBS 3 times for 5 min each time at RT, immersed in 3% H_2_O_2_ for 10 min at RT, washed with PBS 3 times for 5 min each time at RT, incubated with 5% goat serum for 20 min at RT. The slices were incubated with primary antibodies overnight at 4 ˚C. The antibodies included mouse anti- Iba1 / -vascular endothelial growth factor (VEGF) / -platelet endothelial cell adhesion molecule (CD31) (Santa Cruz, dilution ratio: 1:200) and rabbit anti- Arg1 (CST) / -iNOS (Bioworld) / -laminin (Boster) (dilution ratio: 1:100). The slices were washed with PBS 3 times for 5 min each time at RT, and incubated with the appropriate biotinylated secondary antibody (Invitrogen) for 30 min at 37 ˚C, then washed again with PBS 3 times for 5 min each time at RT, and incubated with streptavidin–biotin complex (Biosharp) for 20 min at RT. After that, diaminobenzidine (DAB, CST) was used to detect immune-positive products. Hematoxylin is adopted to stain the nuclei. Each sample was randomly photographed with a light microscope (Olympus, Japan) at a 400-fold magnification around the lesion for 5 fields. Then the images were analyzed with Image-Pro Plus 6 software to obtain the number of immunopositive structures/cells or the optical density of immunopositive products.

For IF, the brain sections were incubated with primary antibodies including rabbit anti-β-tubulin (Beyotime) /—MBP (Abcam) (dilution ratio: 1:200) and mouse anti- SYP (Santa Cruz) /—PSD-95 (CST) (dilution ratio: 1:200) overnight at 4 ˚C, and washed with PBS 3 times for 5 min each time at RT, then exposed to Alexa Fluor 594-conjugated goat anti- rabbit / mouse IgG (Invitrogen) (dilution ratio: 1:500) for 1 h at RT. The slices were washed with PBS 3 times for 5 min each time at RT, and 4,6-diamino-2-phenyl indole (DAPI) was used to stain the cell nuclei. Each sample was randomly photographed with a fluorescence microscope (ZEISS, Germany) at a 400-fold magnification around the lesion for 5 fields. Then the images were analyzed with ImageJ software to acquire the fluorescence intensity of immunopositive products. The counting and optical density of IHC and fluorescence intensity of IF were scored by an investigator in blind fashion.

### Enzyme-Linked Immunosorbent Assay (ELISA)

After ICH in the patients, the contents of NSE and myelin basic protein (MBP) in the serum are closely related to the severity of the brain injury [69], so the concentrations of the two proteins in the experimental rat serum were tested via the colorimetric ELISA method. On day 7 post-treatment, the blood of the animals was collected to isolate the serum, and the latter was used to examine the levels of NSE and MBP according to the instructions of the NSE (Jiancheng Bioengineering Company) and MBP (Novus Biologicals) kits. The samples and different concentrations of standard products were added to the corresponding wells at 100 μl/well and incubated at RT for 120 min. Biotinylated antibody was added at 100 μl/ well and incubated at RT for 60 min. Streptavidin labeled with horseradish peroxidase was added at 100 μl/ well and incubated at RT for 20 min away from light. The plates were washed with a microplate washer five times between adjacent steps above. TMB solution was added at 100 μl/well and incubated at RT for 15–20 min away from light. Termination solution was added at 50 μl/ well and the absorbance was measured at 450 nm by a microplate reader (Multiskan Go, Finland). The standard curve was drawn with the standard substance concentration. The corresponding concentrations of the samples were calculated according to the absorbance value of the sample and the standard curve.

### Western blotting

After 7 days of treatment, three rats in each group were sacrificed. The ipsilateral caudate nuclei of their brains were immediately dissected and stored in liquid nitrogen. Subsequently, the tissues were grinded to pieces, and added to radioimmunoprecipitation assay (RIPA) lysis buffer (Beyotime) for lysis and homogenization, then centrifuged to take the supernatant. Total proteins in the supernatant were separated using gel electrophoresis, and the protein bands in the gel were transmigrated to polyvinylidene fluoride (PVDF) membranes. After treatment with 5% skimmed milk for 30 min at RT, the membranes were incubated with primary antibodies against iNOS (1:500), Arg1 (1:500), and GAPDH (Abcam, 1:10,000) overnight at 4 ˚C, and washed with Tris buffered saline -Tween (TBST) 3 times for 10 min each time at RT. Next, the membranes were placed in HRP-conjugated suitable secondary antibody (Bio-Rad, 1:2000) for 60 min at RT, and washed with TBST 3 times for 10 min each time at RT. The protein signals were detected with a horseradish peroxidase chromogenic kit (Thermo), and the protein bands were quantified using image analysis software (Quantity one) by an individual not involved in this experiment.

### Ultrastructural observation

At the end point of the animal experiment, five rats in each group were sacrificed under deep anesthesia, their brains were taken out and 1 mm^3^ tissue masses were cut from peri-hematoma tissues, then washed with physiological saline and fixed with 3% glutaraldehyde at 4 °C. After serial chemical treatment with 1% Osmium tetroxide, gradient acetone solution, propylene oxide, the samples were embedded in resin and cut into 40 nm sections. After that, the ultra-thin sections were stained with 4% uranyl acetate, and then treated with 0.5% lead citrate. Last, TEM was adopted to observe the ultrastructure of the pathological tissue. All assessments and statistical analyses of the ultrastructure images were conducted by a blind investigator.

### Statistical analysis

All statistical data analysis was conducted utilizing GraphPad Prism (GraphPad Software, Inc.), and the data were shown by the means ± standard deviations. Comparative analysis between the two groups was conducted using the unpaired Student’s t-test. Comparative analysis for the three groups was done by one-way ANOVA, then carried out using Tukey’s multiple comparison test. For all parameters, *P* < 0.05 was viewed as a significant difference.

## Data Availability

Data are available from the corresponding author upon reasonable request.

## Abbreviations

MSCs: Mesenchymal stem cells
ICH: Intracerebral hemorrhage
GDNF: Glial cell line–derived neurotrophic factor
MAP2: Microtubule associated protein 2
NSE: Neuron-specific enolase
MBP: Myelin basic protein
Iba1: Ionized Ca + 2-binding adapter protein 1
iNOS: Inducible nitric oxide synthase
Arg1: Arginase-1
IHC: Immunohistochemistry
IF: Immunofluorescence
VEGF: Vascular endothelial growth factor
CD31: Platelet endothelial cell adhesion molecule
GFAP: Glial fibrillary acidic protein
SYP: Synaptophysin
PSD95: Postsynaptic density protein 95
TEM: Transmission electron microscopy
GFP: Green fluorescent protein
SD: Sprague-Dawley
DMEM: Dulbecco modified eagle medium
PFA: Paraformaldehyde
PBS: Phosphate-buffered saline
H&E: Hematoxylin and eosin
DAB: Diaminobenzidine
DAPI: 4,6-diamino-2-phenyl indole
RIPA: Radio immunoprecipitation assay
PVDF: Polyvinylidene fluoride
TBST: Tris-buffer saline-tween
RT: Room temperature
ELISA: Enzyme-linked immunosorbent assay
^18^F-FDG: ^18^F-fluorodeoxyglucose
